# *Echinococcus granulosus* Genotype and Surgical Outcomes After Cystic Echinococcosis Surgery: A Propensity-Score-Adjusted Retrospective Pilot Cohort Study

**DOI:** 10.3390/tropicalmed11100273

**Published:** 2026-09-29

**Authors:** Mehmet Kadir Bartın, Müge Kara

**Affiliations:** 1 Department of General Surgery, Van Training and Research Hospital, University of Health Sciences, Van 65300, Türkiye; 2 Department of Internal Medicine, Van Training and Research Hospital, University of Health Sciences, Van 65300, Türkiye

**Keywords:** cystic echinococcosis, *Echinococcus granulosus* sensu lato, genotype, propensity-score adjustment, surgical outcome, recurrence, exploratory cohort study

## Abstract

Background: *Echinococcus granulosus* sensu lato comprises several genotypes (G1, G3, G6–G10) that may differ in host range and clinical phenotype, but whether genotype influences localization, cyst size/stage, surgical complexity, or recurrence after cystic echinococcosis (CE) surgery has not been empirically tested; this analysis is accordingly exploratory and hypothesis-generating. Methods: We retrospectively analyzed 80 patients who underwent CE surgery (March 2022–March 2026) with intraoperative genotyping. After excluding eight patients with missing baseline data, a propensity-score-adjusted comparison (age, sex, preoperative albendazole use) compared G1 (*n* = 57) against pooled non-G1 genotypes (*n* = 15) in the 72-patient analytic cohort; because the eligible non-G1 pool was smaller than the matching target, the algorithm retained the entire cohort rather than producing matched pairs. Localization, cyst size/WHO-IWGE stage, surgical difficulty, and recurrence-free survival were compared (effect sizes with 95% CIs alongside *p*-values), with a post hoc sensitivity analysis in the full unmatched cohort (*n* = 80). Results: G1 and non-G1 groups were balanced for age, sex, and antiparasitic pretreatment; cyst multiplicity showed residual imbalance. Intraoperative spillage (33.3% vs. 12.3%; RR 2.71, 95% CI 0.99–7.35) and recurrence (33.3% vs. 17.5%; RR 1.90, 95% CI 0.76–4.72) were numerically higher in non-G1 but not statistically significant (*p* = 0.111, *p* = 0.281); no other domain differed significantly. In the unmatched cohort, spillage reached nominal significance (37.5% vs. 12.5%; *p* = 0.029), while recurrence did not (*p* = 0.077). Conclusions: Non-G1 genotypes showed potentially higher point estimates for spillage and recurrence, but the evidence was imprecise and insufficient to establish a genotype–outcome association; adequately powered, prospectively genotyped, multi-center studies are needed.

## 1. Introduction

Cystic echinococcosis (CE), caused by the larval stage of *Echinococcus granulosus* sensu lato, remains a neglected zoonotic disease with high endemicity in pastoral regions of the Mediterranean basin, Central Asia, and South America [1,2]. Surgery continues to be the mainstay of treatment for large, complicated, or symptomatic cysts [3,4], and postoperative recurrence remains a major determinant of long-term morbidity [4,5].

Molecular studies have shown that *E. granulosus* sensu lato is not a single homogeneous organism but a complex of genetically distinct genotypes (G1, G3, G6, G7, G8, G10), now recognized as comprising at least two species-level groups, *E. granulosus* sensu stricto (G1/G3) and *E. canadensis* (G6/G7/G8/G10). G1 is overwhelmingly the most frequent cause of human CE worldwide, while the remaining genotypes are reported with substantial regional and host-associated variability; G8 and G10, which circulate mainly in sylvatic cervid-carnivore cycles, are only exceptionally documented in human infection [6,7,8].

Whether genotype carries clinically meaningful phenotypic correlates—organ tropism, growth behavior, operative complexity, or recurrence risk—is not well established. Existing evidence is largely limited to small, heterogeneous case series that seldom apply systematic molecular confirmation or account for confounding by baseline demographic or treatment factors [9,10], and genotype prevalence is itself highly skewed toward G1, which complicates direct statistical comparison across genotype categories [6,11].

The World Health Organization Informal Working Group on Echinococcosis (WHO-IWGE) ultrasound staging classification (CE1–CE5) [12,13] and the Clavien–Dindo classification of surgical complications [14] provide standardized frameworks for characterizing, respectively, cyst size/sonographic activity and postoperative morbidity, and are well suited to serve as structured outcome measures in a genotype–outcome analysis.

Because no previous study has prospectively tested genotype as a determinant of surgical or recurrence outcomes in CE, the hypothesis that *E. granulosus* genotype influences localization, cyst size/stage, surgical difficulty, or recurrence is biologically plausible but empirically untested. We therefore explicitly frame the present study as exploratory and hypothesis-generating rather than confirmatory, and we interpret all *p*-values and effect estimates accordingly.

We therefore designed a retrospective cohort analysis, incorporating systematic intraoperative genotyping and propensity-score adjustment to reduce confounding introduced by the marked imbalance in genotype prevalence, to examine the association between *E. granulosus* sensu lato genotype (G1 versus pooled non-G1) and four clinically relevant domains: anatomic localization, cyst size and sonographic stage, surgical difficulty, and recurrence risk after CE surgery.

## 2. Materials and Methods

### 2.1. Study Design and Setting

This is a retrospective, single-center cohort study of consecutive patients who underwent elective or urgent surgery for hepatic or extrahepatic CE [3] between March 2022 and March 2026, with intraoperative sampling of cyst wall/germinal layer material submitted for molecular genotyping. Genotyping targeted two mitochondrial loci, cytochrome c oxidase subunit 1 (*cox1*) and NADH dehydrogenase subunit 1 (*nad1*), using the primer pairs originally described for *Echinococcus* species differentiation (*cox1*: JB3/JB4.5, amplicon ≈ 450 bp; *nad1*: JB11/JB12, amplicon ≈ 471 bp) [15]. PCR amplification used an initial denaturation at 94 °C for 5 min, followed by 35 cycles of denaturation (94 °C, 30 s), annealing (50–55 °C, 30 s) and extension (72 °C, 45 s), with a final extension at 72 °C for 7 min. Amplicons were purified and bidirectionally Sanger-sequenced; resulting sequences were aligned against GenBank reference sequences for *E. granulosus* sensu lato genotypes (G1, G3, G6–G10) using BLAST 2.12 with genotype assigned when nucleotide identity to a single reference genotype was ≥99%. No sample in this cohort showed discordant *cox1*/*nad1* assignment or yielded only a single successfully amplified locus—if this occurred, the sample was pre-specified for exclusion from genotype-based analysis. All procedures were performed under general anesthesia via open laparotomy or thoracotomy according to cyst location; hypertonic saline (20%) was applied as a scolicidal agent to the operative field prior to cyst opening whenever technically feasible, to reduce the risk of anaphylaxis and secondary seeding. Preoperative albendazole, when administered, followed a weight-based regimen (10–15 mg/kg/day in two divided doses) for a minimum of 4 weeks before surgery and was continued postoperatively for 1–3 months at the treating surgeon’s discretion; this exposure was recorded as a binary covariate (preoperative albendazole use) for the propensity-score model (Section 2.4). This study was approved by the Ethics Committee of Health Sciences University Van Training and Research Hospital (protocol code 2022/11-19; approved 24 February 2022); further details are provided in the Institutional Review Board Statement.

### 2.2. Patients, Eligibility, and Exclusion Criteria

The source cohort comprised 80 patients with a confirmed intraoperative genotype result. Three pre-specified exclusion criteria were applied prior to analysis: (i) missing cyst diameter at diagnosis, required for the cyst-size outcome (*n* = 2); (ii) missing WHO-IWGE ultrasonographic stage, required for the same outcome domain (*n* = 2, no overlap with [i]); and (iii) indeterminate recurrence status due to loss to follow-up before recurrence could be radiologically confirmed or excluded, since recurrence-free survival could not be reliably estimated for these patients (*n* = 4). These four patients were excluded outright, rather than administratively censored at last contact, because the absence of confirmed imaging follow-up made it impossible to distinguish true recurrence-free survival from an undetected recurrence; treating them as censored at an uncertain time risked biasing the recurrence-free survival estimate in either direction. A formal comparison of baseline characteristics between excluded and retained patients was not performed given the very small number of excluded patients (*n* = 8 overall, *n* = 4 for the recurrence-related exclusion); we acknowledge the resulting potential for selection bias as a limitation (Section 5). Application of these criteria excluded 8 of 80 patients (10.0%), leaving 72 patients eligible for analysis. The patient flow is summarized in Figure 1.

### 2.3. Genotype Grouping

Among the 72 eligible patients, genotype G1 accounted for 57 cases (79.2%), with the remaining 15 patients (20.8%) distributed across G3 (*n* = 6), G6 (*n* = 6), G7 (*n* = 1), G8 (*n* = 1), and G10 (*n* = 1) (Figure 2). Because individual non-G1 genotype cells were too small to support pairwise statistical comparison, patients were dichotomized a priori into a G1 group and a pooled non-G1 group (G3 + G6 + G7 + G8 + G10), consistent with the pronounced genotype-prevalence skew typical of single-center CE surgical series. This pooled non-G1 category is biologically heterogeneous, spanning both *E. granulosus* sensu stricto (G3) and *E. canadensis* (G6, G7, G8, G10) lineages with distinct host cycles as discussed under “Host Biology and Regional Context” (Section 4); pooling was a pragmatic response to small cell sizes, and the pooled group should not be interpreted as a single, homogeneous biological phenotype.

### 2.4. Propensity-Score-Adjusted Analysis

To reduce confounding arising from the unequal size of the G1 and non-G1 groups, a propensity score (PS) for non-G1 genotype was estimated using multivariable logistic regression [16], with age, sex, and preoperative albendazole used as covariates. These three variables were selected a priori as baseline demographic/treatment-pathway factors plausibly related to referral and management patterns but conceptually distinct from the four outcome domains under study (localization, cyst size/stage, surgical difficulty, recurrence), so as to avoid conditioning on variables downstream of the exposure.

A greedy nearest-neighbor matching algorithm (up to 1:4, without replacement, no caliper) was applied to the estimated logit (PS). Because the eligible G1 pool (*n* = 57) was smaller than the 1:4 target (*n* = 60), all 57 eligible G1 patients were retained, and the resulting cohort (*n* = 72; 57 G1, 15 non-G1) is therefore identical to the eligible cohort in Section 2.2—the algorithm did not exclude any additional patient. Consequently we describe this analysis as a propensity-score-adjusted cohort comparison rather than a matched-pairs analysis. We did not impose a caliper because, given the small non-G1 pool (*n* = 15), any caliper strict enough to materially change the cohort risked excluding non-G1 patients and eliminating the comparison entirely; we therefore report propensity-score diagnostics (standardized mean differences, below) transparently alongside the unmatched result rather than presenting an artificially ‘matched’ analysis. Covariate balance was assessed using the standardized mean difference (SMD), with SMD < 0.10 considered indicative of good balance. Age and preoperative albendazole use were adequately balanced (SMD 0.187 and −0.037, respectively); sex (SMD −0.184) and, more markedly, cyst multiplicity (single versus multiple cysts, not included in the PS model; SMD 0.563) showed residual imbalance, addressed as a limitation and considered when interpreting outcomes that could plausibly be influenced by these factors. Anatomic localization, cyst size, WHO-IWGE stage, and surgical technique were deliberately not added to the propensity-score model despite being baseline-measurable, because they constitute the outcome domains under study (Section 2.5) rather than pre-treatment confounders; including them would condition the model on variables downstream of, or coincident with, the exposure–outcome relationship being tested. Cyst multiplicity does not fall within these four outcome domains and was instead entered as an adjustment covariate in the multivariable regression models (Section 2.7); because this analysis retained the entire eligible cohort without exclusion (see above), the adjustment step could not correct this imbalance regardless of which covariates were specified in the model.

### 2.5. Outcome Definitions

Anatomic localization: Hepatic versus extrahepatic (pulmonary, peritoneal, or splenic) cyst site.

Cyst size and WHO-IWGE stage: Because serial pre-diagnosis imaging was not available in this retrospective design, longitudinal cyst growth rate could not be measured directly. Cyst diameter at diagnosis and WHO-IWGE ultrasonographic stage (dichotomized as CE1–CE3a, early/active, versus CE3b–CE5, advanced/degenerating) are reported as cross-sectional descriptors of cyst size and sonographic activity at the time of diagnosis, not as measures of growth; this remains a methodological limitation discussed in Section 5.

Surgical difficulty indicators: Operative duration, choice of radical (pericystectomy/hepatectomy) versus conservative (cystectomy) technique, intraoperative cyst-content spillage, and Clavien–Dindo grade ≥ II postoperative complications represent distinct clinical constructs and are analyzed and interpreted as four separate indicators rather than as a single validated composite score, since no such validated composite exists for CE surgery; we group them under one heading for narrative convenience only (Section 3.4). Intraoperative spillage was defined as macroscopically visible leakage of hydatid cyst fluid, membranes, or daughter cysts into the operative field or peritoneal/pleural cavity during cyst evacuation or resection, as documented in the operative note.

Recurrence: Time from surgery to radiologically confirmed recurrence, with recurrence-free survival estimated by the Kaplan–Meier method and compared between groups using the log-rank test; patients with indeterminate follow-up status were excluded per Section 2.2. Postoperative surveillance imaging (abdominal ultrasonography, supplemented by contrast-enhanced CT/MRI when clinically indicated) was performed at 3, 6, and 12 months after surgery and annually thereafter; recurrence was defined as a new cystic lesion detected at the operative site or elsewhere on follow-up imaging, meeting WHO-IWGE morphological criteria [12,13], in a patient with no such lesion on the immediate postoperative baseline study.

### 2.6. Statistical Analysis

Continuous variables are summarized as mean ± standard deviation and compared using the independent-samples *t*-test (approximately normal variables, e.g., age) or Mann–Whitney U test (non-normal variables, e.g., cyst diameter, operative duration). Categorical variables are summarized as *n* (%) and compared using the chi-square test or Fisher’s exact test, as appropriate to expected cell counts. Recurrence-free survival was estimated by the Kaplan–Meier method and compared with the log-rank test. Because the non-G1 subgroup is small, we report point estimates as odds ratios (OR) or risk ratios (RR) with 95% confidence intervals alongside *p*-values for the principal between-group comparisons, and we avoid describing *p*-values between 0.05 and 0.10 as ‘approaching significance’; emphasis throughout is on the magnitude and precision of the estimated effect rather than on whether an arbitrary *p* < 0.05 threshold was crossed. A two-sided *p* < 0.05 was considered statistically significant; given the small non-G1 subgroup (*n* = 15), all comparisons should be interpreted as exploratory. As a post hoc sensitivity analysis, the spillage and recurrence comparisons were repeated in the full unmatched cohort (*n* = 80, before exclusion) to assess whether the exclusion step altered the observed association. Analyses were performed in Python 3.11 [17] using pandas [18] for data management, scikit-learn [19] for the logistic propensity-score model, and scipy [20] for hypothesis tests.

### 2.7. Multivariable Regression

To evaluate whether the genotype–spillage and genotype–recurrence associations persisted after adjustment for baseline covariates, multivariable logistic regression models (intraoperative spillage; recurrence, analyzed as a binary endpoint) and a multivariable Cox proportional-hazards model (recurrence-free survival, using the Efron approximation for tied event times) were fitted in the analytic cohort (*n* = 72). All three models adjusted for the same baseline covariates: age, sex, preoperative albendazole use, and cyst multiplicity—variables that are conceptually distinct from the four outcome domains under study (Section 2.5; see also Section 2.4) and therefore appropriate for adjustment rather than propensity-score modeling. Models were estimated by maximum likelihood (Newton–Raphson for the logistic models; direct optimization of the Efron partial likelihood for the Cox model); results are reported as adjusted odds ratios (aOR) or hazard ratios (aHR) with 95% Wald confidence intervals. Given the small non-G1 subgroup (*n* = 15) and the limited number of outcome events relative to the number of covariates, these multivariable models are likely susceptible to overfitting and unstable coefficient estimates; we therefore restricted each model to the same small, pre-specified covariate set rather than a larger one. These analyses are therefore regarded as exploratory adjusted analyses rather than confirmatory sensitivity analyses; formal penalized-regression alternatives (e.g., Firth logistic regression) were not applied in the present study but are noted as a methodological improvement for future, larger studies (Section 5).

## 3. Results

### 3.1. Study Flow and Baseline Characteristics

Of 80 patients with intraoperative genotyping, eight were excluded (two missing cyst diameter, two missing WHO-IWGE stage, four with indeterminate recurrence status), leaving 72 eligible patients (Figure 1). Genotype distribution in the analytic cohort is shown in Figure 2. Because the eligible G1 pool (*n* = 57) was smaller than the 1:4 matching target, the propensity-score algorithm retained the entire eligible cohort of 72 patients (57 G1, 15 non-G1) without further exclusion; per Section 2.4 we present this as a propensity-score-adjusted cohort comparison rather than a matched-pairs analysis. The G1 and non-G1 groups did not differ significantly in age, sex distribution, or preoperative albendazole use; cyst multiplicity remained numerically higher in the G1 group, a residual imbalance discussed in Section 5 (Table 1).

### 3.2. Genotype and Anatomic Localization

Hepatic localization was observed in 66.7% (38/57) of G1 patients versus 73.3% (11/15) of non-G1 patients, with pulmonary involvement in 26.3% versus 13.3% and other extrahepatic (peritoneal/splenic) involvement in 7.0% versus 13.3%, respectively. The distribution of localization did not differ significantly between groups (Table 2).

### 3.3. Genotype and Cyst Size/WHO-IWGE Stage

Mean cyst diameter was numerically larger in the non-G1 group (9.6 ± 5.3 cm) than in the G1 group (8.1 ± 3.5 cm; *p* = 0.523; mean difference 1.5 cm, 95% CI −1.3 to 4.3). Advanced WHO-IWGE stage (CE3b–CE5) was more frequent in the G1 group (50.9%) than in the non-G1 group (20.0%; OR for non-G1 vs. G1 0.24, 95% CI 0.06–0.95); this difference did not reach the pre-specified significance threshold (*p* = 0.064) (Table 3).

### 3.4. Genotype and Surgical Difficulty Indicators

Mean operative duration was 154.2 ± 58.6 min for G1 versus 173.5 ± 91.0 min for non-G1 patients (*p* = 0.608; mean difference 19.3 min, 95% CI −29.2 to 67.8), and radical technique was used in 52.6% versus 53.3% (*p* = 1.000; OR 1.03, 95% CI 0.33–3.22). Intraoperative spillage occurred in 33.3% (5/15) of non-G1 patients versus 12.3% (7/57) of G1 patients (OR 3.57, 95% CI 0.94–13.55; RR 2.71, 95% CI 0.99–7.35); this difference did not reach statistical significance in the primary analytic-cohort analysis (*p* = 0.111). Clavien–Dindo grade ≥ II complications occurred in 20.0% versus 21.1% of non-G1 and G1 patients, respectively (*p* = 1.000; OR 0.94, 95% CI 0.23–3.86). As noted in Section 2.5, these four indicators are reported and interpreted separately rather than as a single composite (Table 4).

### 3.5. Genotype and Recurrence Risk

Over a median follow-up of 32 months (G1) and 46 months (non-G1), recurrence occurred in 33.3% (5/15) of non-G1 patients versus 17.5% (10/57) of G1 patients (OR 2.35, 95% CI 0.66–8.39; RR 1.90, 95% CI 0.76–4.72); this difference did not reach statistical significance (*p* = 0.281). Kaplan–Meier recurrence-free survival at 24 months was 84.0% (G1) versus 73.3% (non-G1), and at 48 months was 76.8% versus 73.3% (Figure 3). The log-rank test did not show a statistically significant difference between groups (χ^2^ = 1.22, *p* = 0.269) (Table 5); the corresponding adjusted hazard ratio from the multivariable Cox model is reported in Section 3.7. Median follow-up differed by 14 months between groups (32 vs. 46 months). The Kaplan–Meier and log-rank methods used here account for differential follow-up through their risk-set construction at each event time, so unequal median follow-up does not, by itself, bias this between-group comparison the way that comparing raw cumulative incidence at a single fixed calendar time would. We cannot, however, exclude a difference in surveillance intensity between groups (for example, closer monitoring after an initially more complex presentation), which could still have increased the opportunity to detect recurrence in the non-G1 group; this is acknowledged as a limitation (Section 5).

### 3.6. Post Hoc Sensitivity Analysis: Full Unmatched Cohort

Because neither the spillage nor the recurrence difference reached significance in the primary analytic-cohort analysis despite the magnitude of the observed difference, both comparisons were repeated in the full unmatched cohort of 80 patients (64 G1, 16 non-G1; i.e., before the exclusion criteria in Section 2.2), to assess whether exclusion had materially altered the observed association (Table 6). In this unmatched cohort, intraoperative spillage was significantly more frequent in the non-G1 group (37.5%, 6/16) than in the G1 group (12.5%, 8/64; OR 4.20, 95% CI 1.20–14.72; RR 3.00, 95% CI 1.21–7.42; *p* = 0.029, Fisher’s exact test). Recurrence was numerically more frequent in the non-G1 group (37.5%, 6/16) than in the G1 group (15.6%, 10/64; OR 3.24, 95% CI 0.96–10.94) but did not reach significance (*p* = 0.077). We do not interpret this nominally significant unmatched-cohort spillage association as evidence of a true genotype effect: its disappearance after exclusion of incomplete-data patients and propensity-score adjustment indicates that the signal was not robust across analytical specifications, and it should be treated with caution rather than emphasized (see Section 4).

### 3.7. Multivariable Regression Analysis

In multivariable logistic regression adjusting for age, sex, preoperative albendazole use, and cyst multiplicity, non-G1 genotype remained associated with numerically higher, but not statistically significant, odds of intraoperative spillage (adjusted OR 3.25, 95% CI 0.81–13.00; *p* = 0.096) and of recurrence (adjusted OR 2.14, 95% CI 0.55–8.40; *p* = 0.274) (Table 7). In the multivariable Cox proportional-hazards model for recurrence-free survival, the adjusted hazard ratio for non-G1 genotype was 1.72 (95% CI 0.56–5.23; *p* = 0.342), consistent in direction and similar in magnitude to the unadjusted log-rank comparison (Section 3.5). None of age, sex, preoperative albendazole use, or cyst multiplicity reached significance in any of the three models (Table 7). As discussed in Section 2.7, these models are likely underpowered and potentially unstable given the small non-G1 subgroup and limited events per variable; we present them here as an exploratory adjusted analysis that is directionally consistent with, but does not statistically confirm, the primary comparisons (Section 3.4 and Section 3.5). The numbers of patients at risk at each follow-up time point are presented in Table 8.

## 4. Discussion

In this propensity-score-adjusted, exploratory pilot cohort, non-G1 genotypes showed a numerically higher rate of intraoperative spillage and recurrence than G1 (spillage: RR 2.71, 95% CI 0.99–7.35; recurrence: RR 1.90, 95% CI 0.76–4.72), but neither outcome reached statistical significance in the pre-specified primary (analytic-cohort) analysis (spillage 33.3% vs. 12.3%, *p* = 0.111; recurrence 33.3% vs. 17.5%, *p* = 0.281; log-rank *p* = 0.269). This illustrates how a small non-G1 subgroup (*n* = 15) limits statistical power. Even a true effect of this magnitude can fail to reach *p* < 0.05 when so few patients are available for comparison, so a non-significant result here should not be read as evidence against a true association.

A post hoc sensitivity analysis using the full, unmatched cohort of 80 patients did show a nominally significant spillage difference (37.5% vs. 12.5%, *p* = 0.029; OR 4.20, 95% CI 1.20–14.72), but this association was attenuated to non-significance after applying the pre-specified exclusion criteria and propensity-score adjustment. Plausible explanations include a genuine reduction in confounding by age, sex, and antiparasitic pretreatment once these were balanced; a loss of statistical power from the smaller analytic sample; or simply the interaction of a modest true effect size with random sampling variability across the two analytic subsets. As noted in Section 3.6, we do not treat the unmatched-cohort signal as evidence of a genotype effect; rather, its disappearance after exclusion and adjustment shows that it was not robust across analytical specifications. This pattern—an unadjusted association that attenuates after confounding adjustment—is a common and important feature of real propensity-score analyses, and its appearance here illustrates why unadjusted and adjusted analyses can legitimately disagree and why the adjusted, pre-specified analysis should be regarded as primary.

Beyond the two outcomes emphasized in this study, no other domain—localization, cyst size/stage, operative duration, radical resection rate, or Clavien–Dindo grade ≥ II complications—differed significantly between groups, and the residual imbalance in cyst multiplicity after adjustment (Section 2.4) further limits confidence in any of the point estimates. Systematic molecular genotyping of resected cyst material nonetheless retains value beyond genotype–outcome comparison: it supports transmission-cycle mapping and zoonotic reservoir surveillance in endemic regions, and, at the individual-patient level, allows discrimination between true recurrence (same genotype as the primary cyst) and probable reinfection (a different genotype), a distinction not achievable through clinical or radiological follow-up alone.

Genotype may also shape the host immune response. Experimental models have shown that *E. granulosus* protoscolex and hydatid-fluid antigens elicit a mixed Th1/Th2 cytokine response that shifts toward a permissive, Th2-dominant profile as chronic infection is established, with the parasite actively modulating nitric-oxide and cytokine pathways to favor its own persistence [8,21]. Whether such immunomodulatory capacity differs quantitatively between G1 and the pooled non-G1 genotypes examined here—and whether this could plausibly contribute to differences in spillage-related peritoneal seeding or recurrence risk—has not been directly tested and remains an important target for future genotype-stratified immunological studies.

The broader epidemiological and clinical literature on *E. granulosus* sensu lato provides additional context for interpreting the genotype-associated patterns observed here. Systematic reviews of genotyping data collected across Europe over the past two decades confirm that G1 remains numerically dominant in human cystic echinococcosis, with G6/G7 and, more rarely, G8/G10, accounting for a persistent minority of cases whose clinical significance is increasingly recognized rather than dismissed as incidental [11]. Contemporary clinical-management reviews likewise emphasize that treatment algorithms for CE—cyst staging, choice between surgical, percutaneous, and medical therapy, and follow-up strategy—were developed largely from sensu stricto (G1/G3) cohorts, so their applicability to non-G1 disease remains an open question that genotype-stratified outcome studies such as the present one are beginning to address [4,5].

Host Biology and Regional Context

Beyond the associations examined here, an emerging literature increasingly supports genotype-dependent differences in the clinical behavior of *E. granulosus* sensu lato. In a comparative Argentine cohort, Debiaggi et al. [9] found that CE caused by the G6 genotype followed a distinct clinical course from *E. granulosus* sensu stricto (G1/G3), including differences in cyst staging and organ distribution, broadly consistent with the numerically higher spillage and recurrence observed for non-G1 genotypes in the present cohort. Such genotype-associated virulence differences have been attributed to variation in germinal-layer growth rate, protoscolex fertility, and laminated-layer composition between sensu stricto and sensu lato lineages, although head-to-head experimental comparisons in humans remain scarce.

Host specificity differs markedly across the *E. granulosus* sensu lato complex and helps explain why non-G1 genotypes remain comparatively rare in human CE. G1/G3 (sensu stricto) circulate predominantly in a domestic dog–sheep/cattle cycle with broad intermediate-host permissiveness, whereas G6/G7 (camel/pig strains) and G8/G10 (*E. canadensis*) are maintained chiefly in cervid–wolf or other sylvatic carnivore–ungulate cycles, with only incidental spillover into domestic dogs and humans [10,22]. A recent report of G6/G7 co-infection in a gray wolf in Turkey illustrates how sylvatic and domestic transmission cycles can overlap geographically, creating local foci where humans may be exposed to non-G1 genotypes despite the overall predominance of G1 [23]. Because these host-cycle differences also correlate with intermediate-host organ tropism and cyst developmental biology, they offer a plausible biological substrate for the localization and cyst-size/stage differences examined in Section 3.2 and Section 3.3, even though neither reached significance in the present sample.

Regional epidemiological patterns further contextualize the genotype distribution observed in this cohort. Global burden-of-disease modeling projects that CE incidence, and by extension its genotype composition, will remain concentrated through 2036 in pastoral regions of Central Asia, the Mediterranean basin, and South America, with slow overall decline offset by persistent hotspots [1], and a disproportionate burden continues to fall on children and adolescents in endemic low- and middle-income settings [2]. Within Europe and neighboring regions, genotype surveys continue to show G1 predominance with a persistent minority of G6/G7/G8/G10 infections concentrated in specific ecological niches [11], a pattern broadly mirrored by the 79.2%/20.8% G1/non-G1 split observed in the present single-center Turkish cohort.

At the molecular level, mitochondrial genome analyses indicate that G8 and G10 harbor greater intra-genotype genetic diversity than previously appreciated, with phylogeographic evidence suggesting a northeastern Asian origin for G10 and subsequent dispersal alongside cervid and canid host movements into Europe [24,25]. Such ongoing genetic diversification could, in principle, be accompanied by adaptive shifts in growth rate, host range, or virulence that would be directly relevant to genotype–outcome studies such as this one; the present pilot cohort, however, characterized genotype only at the G-level by mitochondrial cox1/nad1 typing and cannot address within-genotype (sub-lineage) variation, which remains an important direction for future work.

## 5. Limitations

This study has several limitations. First, the retrospective, single-center design and the small non-G1 subgroup (*n* = 15) substantially limit statistical power and preclude genotype-specific (rather than pooled non-G1) comparisons; the possibility of a type II error cannot be excluded for any outcome domain. Second, true longitudinal cyst growth rate could not be measured directly because serial pre-diagnosis imaging was not available, and cyst diameter and WHO-IWGE stage at a single time point were used as cross-sectional descriptors of cyst size, which may not fully capture growth dynamics. Third, despite propensity-score adjustment for age, sex, and preoperative albendazole use, residual imbalance in sex and, more markedly, cyst multiplicity, persisted and could not be fully corrected given the limited size of the non-G1 pool; this residual confounding should be considered when interpreting all reported outcomes. Fourth, because the eligible G1 pool was smaller than the 1:4 matching target, the propensity-score algorithm did not exclude any additional patients beyond the initial eligibility criteria, so this analysis is a propensity-score-adjusted comparison rather than a true matched-pairs analysis, and effect estimates should be interpreted with this in mind. Fifth, recurrence ascertainment depended on the completeness and interval of imaging follow-up, and under-ascertainment of asymptomatic recurrence cannot be excluded despite exclusion of patients with indeterminate follow-up status. Sixth, the four patients excluded for indeterminate recurrence status were not formally compared with the retained patients on baseline characteristics, given their very small number; we cannot exclude the possibility that these exclusions introduced a selection or informative-censoring bias into the recurrence analysis. Seventh, median follow-up differed by 14 months between groups (32 months G1 vs. 46 months non-G1); although the Kaplan–Meier/log-rank methods used here are designed to accommodate unequal follow-up through risk-set construction, we cannot exclude a difference in surveillance intensity between groups that could have increased the opportunity to detect recurrence in the more closely followed group. Eighth, the pooled non-G1 category combines biologically heterogeneous lineages (*E. granulosus* sensu stricto G3, and *E. canadensis* G6/G7/G8/G10) that should not be interpreted as a single phenotype (Section 2.3). Ninth, the multivariable regression models (Section 2.7) include a limited number of events relative to covariates and may be unstable; penalized-regression approaches were not used in the present study. Tenth, this single-center cohort used one mitochondrial genotyping protocol without an external quality-assurance exchange; broader validation would strengthen generalizability.

## 6. Conclusions

In this exploratory, propensity-score-adjusted pilot cohort, non-G1 genotypes showed a numerically elevated rate of intraoperative spillage and recurrence compared with G1, but neither outcome reached statistical significance in the pre-specified primary analysis, and the nominally significant unmatched-cohort spillage signal (Section 3.6) was not robust to exclusion and adjustment. The most defensible conclusion is that non-G1 genotypes showed potentially higher point estimates for intraoperative spillage and recurrence, but the evidence was imprecise and insufficient to establish a genotype–outcome association. Adequately powered, prospectively genotyped, multi-center cohorts would be required to confirm whether a genuine association between genotype and either intraoperative spillage or recurrence risk exists.

## Figures and Tables

**Figure 1:**
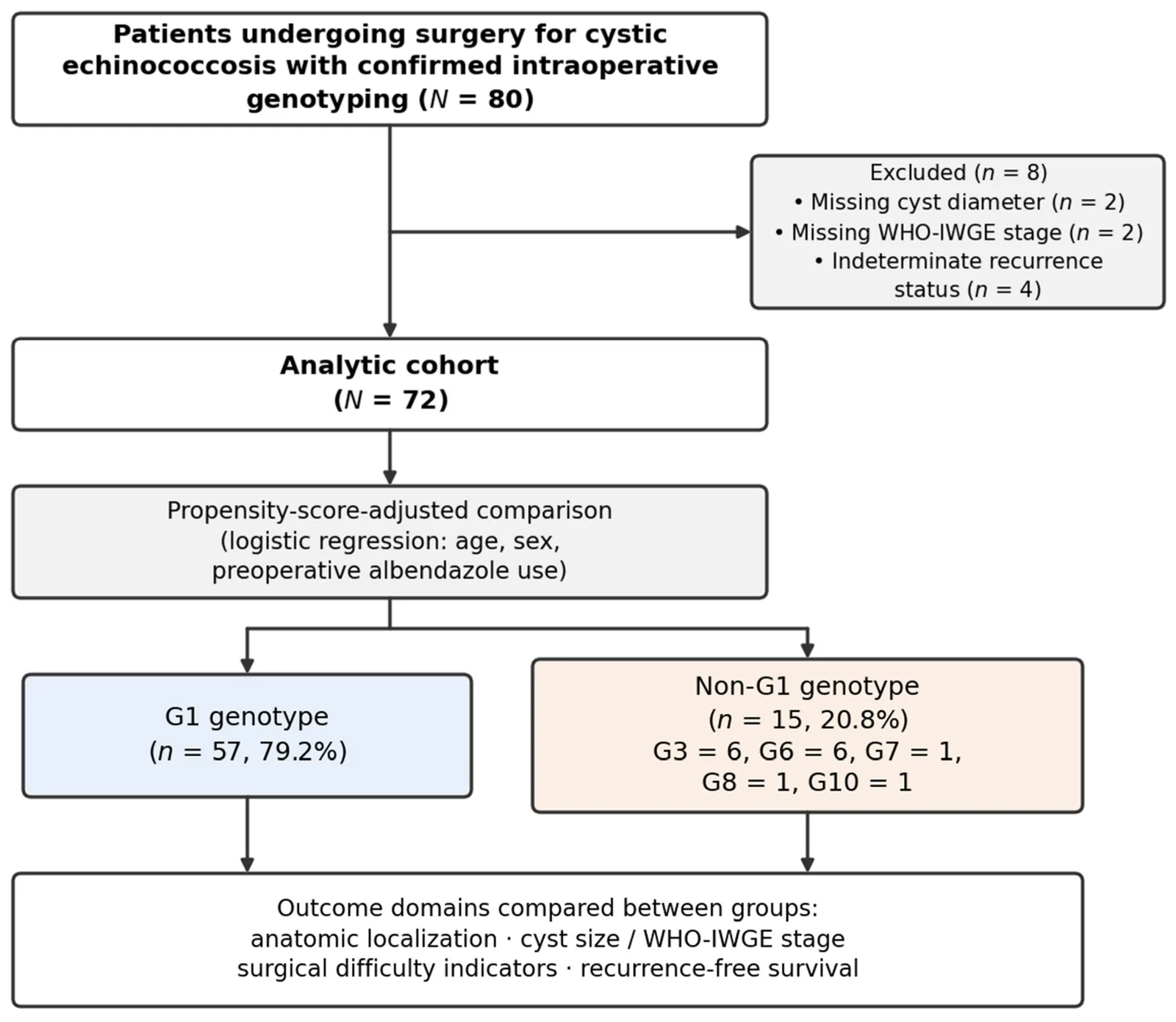
Patient flow from the source cohort to the analytic cohort.

**Figure 2:**
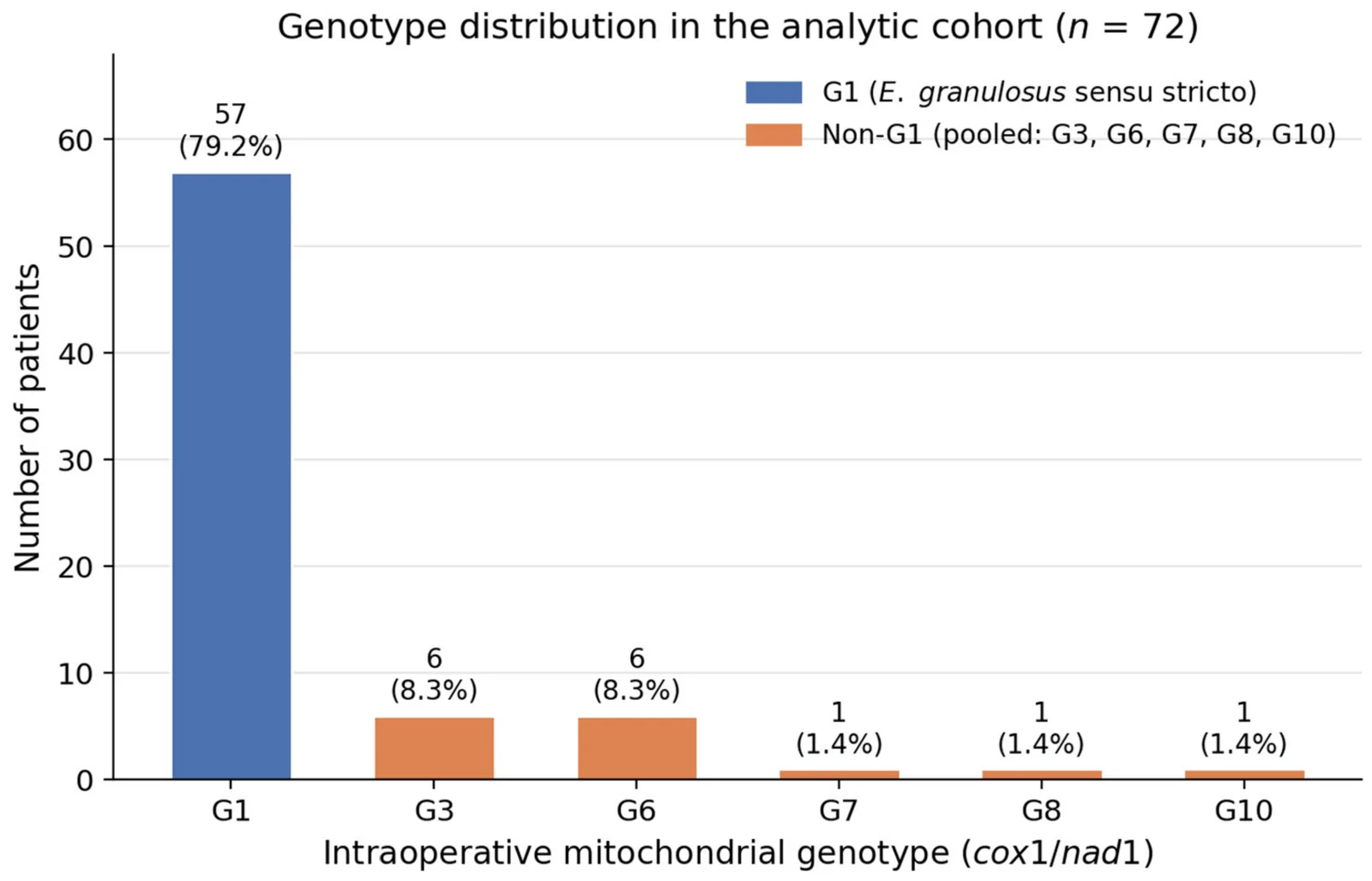
Genotype distribution in the analytic cohort (*n* = 72), supporting Section 2.3.

**Figure 3:**
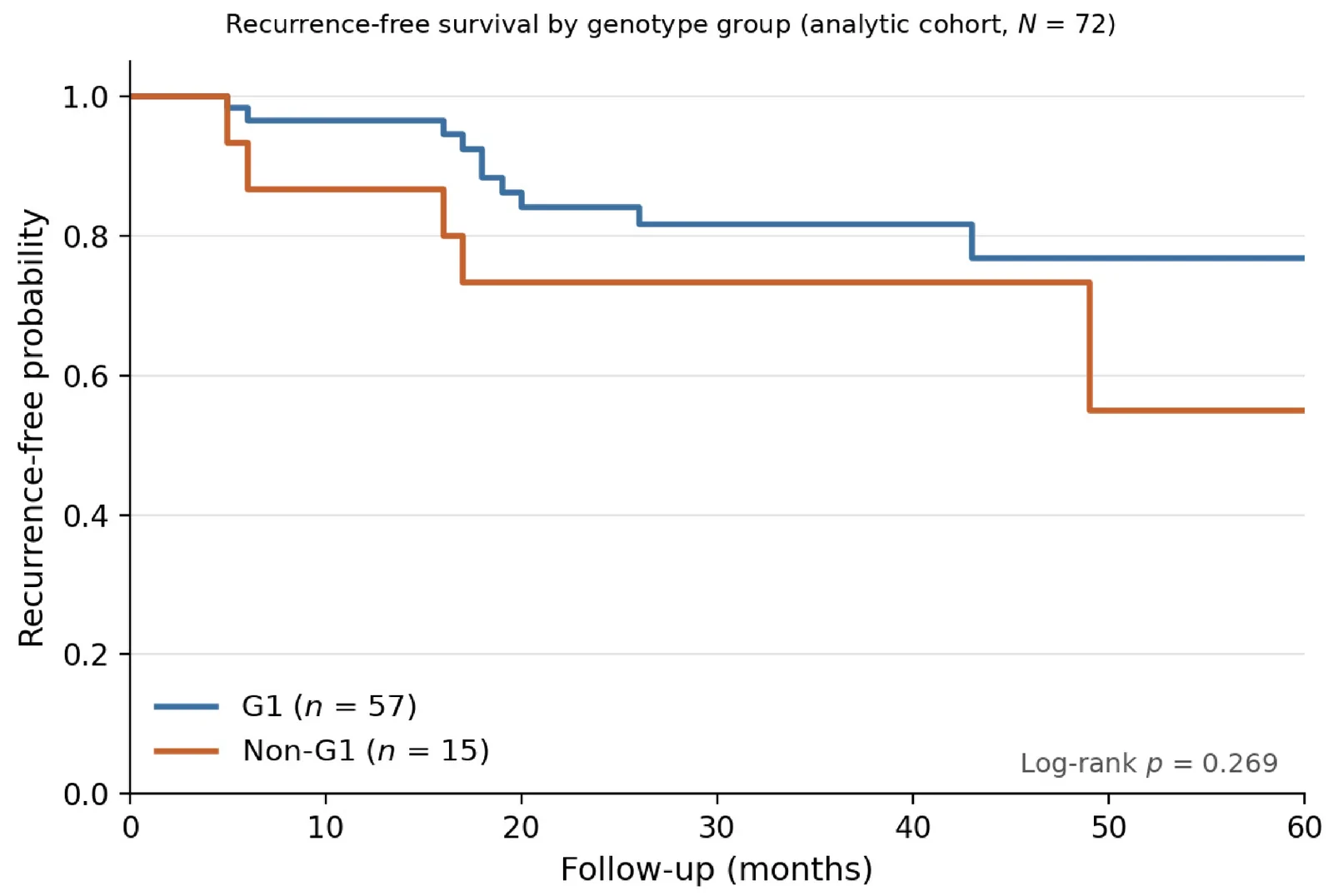
Kaplan–Meier recurrence-free survival by genotype group in the analytic cohort (*N* = 72). Log-rank *p* = 0.269.

**Table 1:** Baseline characteristics of the analytic cohort by genotype group.

Characteristic	G1 (*n* = 57)	Non-G1 (*n* = 15)	*p*	SMD	Effect Estimate (95% CI)
Age, years, mean ± SD	43.0 ± 16.7	40.1 ± 14.3	0.541	0.187	Mean diff −2.9 (−11.3 to 5.5)
Male sex, *n* (%)	29 (50.9)	9 (60.0)	0.574	−0.184	OR 1.45 (0.46–4.60)
Multiple cysts, *n* (%)	21 (36.8)	2 (13.3)	0.121	0.563 *	OR 0.26 (0.05–1.28)
Preop. albendazole, *n* (%)	37 (64.9)	10 (66.7)	1.000	−0.037	OR 1.08 (0.32–3.60)

SMD, standardized mean difference (|SMD| < 0.10 indicates good balance). Because the eligible G1 pool (*n* = 57) was smaller than the 1:4 target, the analytic cohort equals the eligible cohort (Section 2.4). * Residual imbalance not corrected by adjustment, discussed as a limitation (Section 5). *p*-values: *t*-test (age), Fisher’s exact test (categorical variables). Effect estimates (odds ratio for binary variables, mean difference for age) with 95% CI are crude, unadjusted values computed from the reported *n*(%)/mean ± SD, non-G1 vs. G1.

**Table 2:** Genotype group and anatomic localization.

Localization	G1 (*n* = 57)	Non-G1 (*n* = 15)	*p*	Effect Estimate (95% CI)
Liver, *n* (%)	38 (66.7)	11 (73.3)		
Lung, *n* (%)	15 (26.3)	2 (13.3)		
Peritoneal/splenic, *n* (%)	4 (7.0)	2 (13.3)	0.856 ^a^	OR 1.38 (0.39–4.90)

^a^ Chi-square test, hepatic vs. extrahepatic localization. Hepatic vs. extrahepatic, non-G1 vs. G1: RR 1.10 (95% CI 0.77–1.57).

**Table 3:** Genotype group and cyst size/WHO-IWGE stage.

Indicator	G1 (*n* = 57)	Non-G1 (*n* = 15)	*p*	Effect Estimate (95% CI)
Cyst diameter, cm, mean ± SD	8.1 ± 3.5	9.6 ± 5.3	0.523 ^a^	Mean diff 1.5 (−1.3 to 4.3)
Advanced stage (CE3b–CE5), *n* (%)	29 (50.9)	3 (20.0)	0.064 ^b^	OR 0.24 (0.06–0.95)

^a^ Mann–Whitney U test. ^b^ Chi-square test. Effect estimates are non-G1 vs. G1.

**Table 4:** Genotype group and surgical difficulty indicators.

Indicator	G1 (*n* = 57)	Non-G1 *(n* = 15)	*p*	Effect Estimate (95% CI)
Operative duration, min, mean ± SD	154.2 ± 58.6	173.5 ± 91.0	0.608 ^a^	Mean diff 19.3 (−29.2 to 67.8)
Radical technique, *n* (%)	30 (52.6)	8 (53.3)	1.000 ^b^	OR 1.03 (0.33–3.22)
Intraoperative spillage, *n* (%)	7 (12.3)	5 (33.3)	0.111 ^b^	OR 3.57 (0.94–13.55)
Clavien–Dindo ≥ II, *n* (%)	12 (21.1)	3 (20.0)	1.000 ^b^	OR 0.94 (0.23–3.86)

^a^ Mann–Whitney U test. ^b^ Fisher’s exact test. Effect estimates are non-G1 vs. G1.

**Table 5:** Genotype group and recurrence outcome.

Recurrence Outcome	G1 (*n* = 57)	Non-G1 (*n* = 15)	*p*	Effect Estimate (95% CI)
Recurrence events, *n* (%)	10 (17.5)	5 (33.3)	0.281 ^a^	OR 2.35 (0.66–8.39)
Median follow-up, months	32	46		
Recurrence-free at 24 mo, %	84.0 (70.9–97.1)	73.3 (45.9–100.0)		
Recurrence-free at 48 mo, %	76.8 (53.9–99.7)	73.3 (40.5–100.0)	log-rank 0.269	aHR 1.72 (0.56–5.23) †

† Adjusted hazard ratio from the multivariable Cox model (Section 3.7), shown for reference; unadjusted HR/CI could not be back-calculated from the summary Kaplan–Meier data alone. 95% CIs for the 24- and 48-month recurrence-free survival percentages are approximate; they were calculated using a normal (Wald) approximation, *S*(*t*) ± 1.96√[*S*(*t*)(1 − *S*(*t*))/*n*(*t*)], with *n*(*t*) taken as the number of patients at risk at that time. This is a simplified pointwise approximation rather than the full Greenwood estimator, since per-interval event/censoring counts were not further decomposed, and is intended to convey indicative precision rather than an exact stepwise confidence band. ^a^ Fisher’s exact test.

**Table 6:** Post hoc sensitivity analysis: spillage and recurrence in the full unmatched cohort.

Outcome (Full Unmatched Cohort, *n* = 80)	G1 (*n* = 64)	Non-G1 (*n* = 16)	*p*	Effect Estimate (95% CI)
Intraoperative spillage, *n* (%)	8 (12.5)	6 (37.5)	0.029 ^a^	OR 4.20 (1.20–14.72)
Recurrence, *n* (%)	10 (15.6)	6 (37.5)	0.077 ^a^	OR 3.24 (0.96–10.94)

^a^ Fisher’s exact test. Post hoc sensitivity analysis on the full cohort before exclusion (Section 2.2); see Section 3.6 and Section 4 for interpretation. Effect estimates are non-G1 vs. G1.

**Table 7:** Multivariable regression models for intraoperative spillage, recurrence (binary), and recurrence-free survival, analytic cohort (*n* = 72).

Covariate	Spillage, aOR (95% CI), *p*	Recurrence, aOR (95% CI), *p*	Recurrence-Free Survival, aHR (95% CI), *p*
Non-G1 genotype (vs. G1)	3.25 (0.81–13.00), *p* = 0.096	2.14 (0.55–8.40), *p* = 0.274	1.72 (0.56–5.23), *p* = 0.342
Age, per 10 years	0.92 (0.60–1.40), *p* = 0.696	0.71 (0.47–1.08), *p* = 0.109	0.71 (0.49–1.02), *p* = 0.060
Male sex	1.16 (0.31–4.26), *p* = 0.827	1.83 (0.52–6.38), *p* = 0.345	1.40 (0.45–4.32), *p* = 0.560
Preoperative albendazole	1.84 (0.42–8.03), *p* = 0.417	2.66 (0.63–11.27), *p* = 0.184	2.25 (0.61–8.34), *p* = 0.224
Multiple cysts (vs. single)	0.75 (0.16–3.41), *p* = 0.708	0.98 (0.25–3.82), *p* = 0.982	1.07 (0.34–3.36), *p* = 0.903

aOR, adjusted odds ratio (logistic regression); aHR, adjusted hazard ratio (Cox proportional-hazards regression, Efron approximation); CI, confidence interval. Recurrence is modeled here as a binary endpoint for the logistic model and as a time-to-event endpoint for the Cox model; see Section 2.7 for covariate rationale and for why these analyses are regarded as exploratory rather than confirmatory.

**Table 8:** Number at risk, by genotype group.

Months from Surgery	0	12	24	36	48	60
G1, number at risk	57	45	30	24	13	5
Non-G1, number at risk	15	11	10	8	7	3

Number of patients remaining under observation at each landmark time.

## Data Availability

The data supporting this study’s findings are available on request from the corresponding author. The data are not publicly available due to privacy or ethical restrictions.
